# Characteristics of HCV positive patients in an Italian urban psychiatric unit

**DOI:** 10.1186/1745-0179-2-26

**Published:** 2006-10-01

**Authors:** Michele Raja, Antonella Azzoni, Daniela Pucci

**Affiliations:** 1 Servizio Psichiatrico di Diagnosi e Cura, Ospedale Santo Spirito, Rome, Italy; 2 Dipartimento di Scienze della Salute Pubblica, University "La Sapienza", Rome, Italy

## Abstract

**Objectives:**

1) to assess the prevalence of hepatitis C virus (HCV) infection in a population of acute psychiatric in-patients; 2) to find out relationships between HCV comorbidity and clinical features of psychiatric patients.

**Methods:**

Prospective observational study in a 6-year period.

**Results:**

2396 cases (1492 patients) were admitted in the considered period. Forty-two patients (2.8%) were affected by HCV infection. HCV infection was more frequent in patients with less years of education, lower social class, lower last year best Global Assessment of Functioning score, more hostile or violent behavior in hospital, with a lifetime history of previous suicide attempt, and with substance-related disorders.

**Conclusion:**

HCV infection in psychiatric patients constitutes a major threat to the health of psychiatric patients and is related with unfavorable social background, worse global functioning, hostile or violent behavior, substance-related disorders. It appears also to be a significant risk of suicidal behavior.

## Background

Patients with comorbid conditions probably represent majority of subjects affected by mood disorders or schizophrenia [1]. Comorbid medical diseases may cause or worsen psychiatric disorders and have a major impact on the medical treatment of patients [2-4]. Nearly 50% of medical comorbid diseases are not diagnosed in chronic psychiatric outpatients [5]. Also in psychiatric in-patients, medical diseases are often not diagnosed [6]. Psychosis may impair the patient's capacity to recognize or describe emerging medical illness. Consequently, psychiatric patients often receive inadequate care [1]. Numerous studies have demonstrated mortality rates that are at least twice as high among persons with severe mental illness, with a life expectancy ten years less than that of the general population. Some authors have attributed these poor health outcomes to clients' lack of regular medical care [7-12].

Hepatitis C is a silent disease, with symptoms developing an average of 20 years after infection. Around 85% of persons infected with hepatitis C develop lifelong chronic infection. Approximately 20% of chronically infected persons will develop cirrhosis, and approximately 3% will develop hepatocellular carcinoma [13]. Hepatitis C is the most common chronic blood-borne infection in Italy as well as in the United States, with an estimated prevalence of 1%–2%. The prevalence of HCV infection varies by geographical region [14,15], is higher among men than women [16], and may be very high in special populations. Population-based studies indicate that 40% of chronic liver disease is HCV related [17]. People with severe mental illness appear to have a higher risk of HCV for a variety of reasons, including elevated rates of injection drug use, multiple, high-risk sexual partners, infrequent use of condoms, a tendency to trade sex for material gain, and engagement in sexual activity while using psychoactive substances [18]. Several psychiatric disorders, including schizophrenia, depressive disorders, psychosis, bipolar disorder, anxiety disorders, were found more frequent among HCV-infected veterans compared with those who were not infected [19].

Klinkenberg et al [20] found a prevalence rate of 30% among homeless persons with co-occurring severe mental illness and substance use disorders. Serum anti-HCV antibodies were detected in 6.7% in institutionalized psychiatric patients [21]. Psychiatric disorders were present in 60% of 206 consecutive patients with HCV infection attending a Veteran Medical Center [22]. An American multisite study of blood-borne infections among persons with severe mental illness found a prevalence of hepatitis C of 19.6 percent, 11 times the overall population rate [23].

While little is known about hepatitis C outcome among the severely mentally ill, it is likely to be worse because of lack of access to and compliance with health care and further worsened by hepatic damage due to substance use. Moreover, since most psychotropic drugs are hepatically metabolized, chronic hepatitis C may complicate pharmacotherapy in this population. The high prevalence of the virus in this population also places mental health care workers at risk for infection, many of whom may be unfamiliar with universal precautions protocol [24]. All that makes HCV infection a first rank problem in the management of psychiatric patients.

The aims of the present naturalistic study were: 1) to assess the prevalence of HCV infection in a population of acute psychiatric in-patients; 2) to find out relationships between HCV comorbidity and clinical features of psychiatric patients.

## Methods

The study was carried out at a12 bed PICU of a general hospital with a catchment area of about 210.000 inhabitants. Admissions exclude persons under age 18. As the hospital is in the center of Rome, near St. Peter's Basilica, we also accept foreign patients with different backgrounds. We do not think our population of patients to be unique if compared to psychiatric patients in general. The patients examined were all those discharged in a six-years period. The following data were ascertained for each patient: sex, age, diagnosis, commitment, length of hospitalization, psychopharmacological treatment on admission and on discharge. Final longitudinal best-estimate diagnoses (DSM-IV-TR) were generated by consensus of the authors. The authors have been cooperating in the assessment of the patients admitted to the PICU in the last ten years and have shown a high inter-rater reliability. In as many patients as possible, as part of clinical routine, we registered years of education, age at the onset of the disorder, history of suicide attempts, and assessed clinical conditions by the Brief Psychiatric Rating Scale (BPRS), including 24 items rated from 1 to 7, the Global Assessment of Functioning (GAF) scale, and the Clinical Global Impression (CGI). Social class was rated using an original scale that considers the years of education and the employment status of the patient and of the head of his/her family, and the residence of the patient (1–5 point scoring system for each item, range of total score: 5–25). We used a modified version of the Morrison's scale [25] to rate patients' highest level of hostile or violent behavior during hospitalization. No distinction was made in the analyses between alcohol/drug abuse or dependence. The base number of patients for each rating scale is shown in the freedom of degree of the statistics reported in Table n. 2. All patients were checked for HCV antibody. Patients underwent laboratory routine in the early morning of the first week-day after their admission. The interval between patients' admission and laboratory routine may have been >48 hours long (in the rare case of two consecutive holiday days). As a consequence, very few cases may have left the hospital before undergoing laboratory routine. Diagnostic comparisons were between persons with the primary psychiatric diagnosis of schizophrenia, schizoaffective disorder, bipolar disorder, unipolar depression, and other diagnoses. Comparison were also made between participants with and without a primary or secondary diagnosis of substance-related disorder. The χ^2 ^test was used to analyze categorical variables. T-test was performed for continuous variables. Although many considered continuous variables are not really interval, we chose to use T-test because of the high number of examined subjects. All p values were two tailed, and statistical significance was set at p < 0.05. Logistic regression is the statistical method of choice for analyzing the effects of independent variables on a dichotomous dependent variable. Multivariate logistic regression has been used to investigate the effect of different risk factors such as any substances abuse, previous suicides attempts, years of education, Morrison Scale on the risk of infection with hepatitis C.

**Table 1:** Psychiatric diagnoses of the 1492 patients

Diagnosis	N° of cases (%)
Schizophrenia	134 (9.0%)
Schizoaffective disorder	134 (9.0%)
Bipolar disorder *mania*	232 (15.6%)
Bipolar disorder *depression*	62 (4.2%)
Bipolar disorder *mixed*	214 (14.3%)
Unipolar depression	89 (6.0%)
Dysthymic disorder or depression NOS	45 (3.0%)
Psychotic disorder NOS	331 (22.2%)
Delusional disorder	14 (0.9%)
Obsessive-compulsive disorder	8 (0.6%)
Dissociative disorders	20 (1.3%)
Alcohol or Substance related disorder	26 (1.7%)
Personality disorder	35 (2.3%)
behavioral misconduct related with Mental retardation	36 (2.4%)
behavioral misconduct related with Dementia	9 (0.6%)
Delirium, Mood or Psychotic disorder due to general medical condition	16 (1.1%)
Asperger's disorder	5 (0.3%)
Eating disorders	5 (0.3%)
Other diagnoses	81 (5.4%)

**Table 2:** Continuous variables in patients with and without hcv infection

Variable	HCV infection	No HCV infection	T	df	P
**Age (years)**	38.6 (± 11.3)	42.1 (± 14.6)	1.515	1480	.130
**Hospitalization length (days)**	13.6 (± 12.0)	11.1 (± 12.7)	-1.261	1476	.207
**Education (years)**	9.2 (± 2.8)	10.8 (± 4.0)	2.070	898	.039*
**Social class**	12.1 (± 3.4)	14.4 (± 4.3)	2.873	886	.004*
**Current GAF**	21.6 (± 4.9)	23.6 (± 7.6)	1.326	910	.185
**Last year best GAF score**	42.9 (± 15.1)	50.5 (± 15.1)	2.641	874	.008*
**Age at the onset of the disorder**	23.4 (± 13.4)	29.1 (± 13.5)	1.909	736	.057
**BPRS total score**	61.0 (± 14.9)	58.7 (± 13.3)	-0.899	883	.369
**Morrison**	2.1 (± 2.8)	1.2 (± 1.9)	-3.053	1470	.002*
**CGI**	5.8 (± 0.6)	5.7 (± 0.6)	0.945	996	.345

BPRS: Brief Psychiatric Rating Scale; GAF: Global Assessment of Functioning Scale; CGI: Clinical Global Impression.

We collected all the data as part of our clinical routine. Consensus was not asked to the patients for the use of the anonymous epidemiological data. As the main purpose of the study was descriptive in nature, we did not specify hypotheses related to the study objectives in advance.

## Results

In the considered period, 2396 cases, 1067 men (44.5%) and 1329 women (55.5%) were admitted to the PICU. Involuntary admissions were 604 (25.2%). Patients' mean age was 41.9 (± 14.1) years, mean years of education were 10.7 (± 3.9). Ethnic background was Caucasian in 98% of cases. The most frequent psychiatric diagnoses are shown in Table n. 1. Since many patients have been admitted more than once in the period of study, with possible bias in the distribution of medical comorbidity, we decided to examine only the first admission for each patient in the considered period, although the observations in the following admissions could have been more accurate. Overall, we found that, among the 2396 cases, 1492 patients have been admitted. Forty-two of them (2.8%) were HCV seropositive. None of them had been treated in the past or was treated with interferon at the time of the study. Among the five major groups of primary psychiatric diagnosis of the 1492 patients, the prevalence of HCV infection was not significantly different: schizophrenia (6/136, 4.4%), schizoaffective disorder (6/143, 4.2%), bipolar disorder (10/534, 1.9%), unipolar depression (1/89, 1.1%), other diagnoses (19/571, 3.3%) [χ^2 ^= 5.282, fd = 4, p = .260]. Between patients with and without HCV infection, there was no significant difference in length of hospitalization, age at the onset of the disorder, in the scores of current GAF, BPRS total, CGI (see Table n. 2), in the percentage of patients treated with antipsychotics, benzodiazepines, valproate, lithium, and in the mean dosage utilized (data not shown). HCV infection was more frequent among patients who received a primary or secondary diagnosis of any substance-related disorder, alcohol-related disorder, cannabis-related disorder, cocaine-related disorder, opioid-related disorder (see Table n. 3). HCV infection tended to be more frequent in men (25/653, 3.8%) than in women (17/839, 2.0%), but the difference failed to reach the level of significance [χ^2 ^= 3.726, fd = 1, p = .054]. Furthermore, patients with HCV infection had less years of education, were of lower social class, received lower last year best GAF and Morrison's scale scores (see Table n. 2). HCV infection was more frequent among the patients with a previous suicide attempt (14/227, 6.2%) than among the patients without previous suicide attempt (12/639, 1.4%) [χ^2 ^= 9.161; fd = 1;p = .002]. Reliable history of previous suicide attempt was unavailable for 625 (41.9%) patients, most of whom were early transferred to other PICUs for overcrowding of our PICU or for administrative reasons. The regression (Table n. 4) shows a significant effect of previous suicide attempts (p = 0.01; OR = 2.87) and substances abuse (p << 0.05; OR = 5.31) on the risk of HCV infection. The association with years of education is not significant (p > 0.05) but there is an indication of a trend: patients who attended high school has a lower risk (OR = 0.57) than patients who attended only elementary school and the Odds ratio is 0.34 for patients with university education. The risk of having hepatitis C is 2.51 times higher in patients with a score between 8 and 9 on the Morrison Scale.

**Table 3:** Categorical variables in patients with and without hcv infection

Variable	HCV infection	No HCV infection	χ^**2**^	df	P
**Gender (M/F)**	25 (59.5%)/17 (40.5%)	628 (43.3%)/822 (56.7%)	3.726	1	.054
**Previous suicide attempt (Y/N)**	14 (33.3%)/12 (28.6%)	213 (14.7%)/627 (43.2%)	9.161	1	.002*
**Any alcohol or substance use disorder**	27 (64.3%)	221 (15.2%)	67.348	1	.000*
**Alcohol use disorder**	13 (31.0%)	142 (9.8%)	17.423	1	.000*
**Cannabis use disorder**	6 (14.3%)	81 (5.6%)	4.153	1	.042*
**Cocaine use disorder**	7 (16.7%)	31 (2.1%)	29.106	1	.000*
**Opioid use disorder**	12 (28.6%)	18 (1.2%)	141.178	1	.000*

**Table 4:** Logistic regression

Independent variables	z	p	OR	Confidential interval 95%
Previous suicide attempt	2,47	0,01*	2,87	1.24 – 6,61
Substance abuse	3,92	0,0001*	5,31	2,31 – 12,22
Scolarity (High school)	-1,24	0,21	0,57	0,23 – 1,39
Scolarity (University years)	-1,37	0,16	0,34	0,08 – 1,57
Morrison scale (1–7)	0,39	0,69	1,19	0,50 – 2,84
Morrison scale (8–9)	-9,5	0,26	2,51	0,49 – 12,82

## Discussion

The strengths of the study were its large sample size and the high number of socio-demographic and clinical variables assessed in most patients. Despite these strengths, we must acknowledge several significant limitations. First, as in all non-epidemiological studies, the sample may not be representative. Hospital-based samples are not representative of the spectrum of the disorder found in the general population or in outpatient samples. Second, history of previous suicide attempts was not available in nearly 40% of the patients. However, we have no reason to suspect that these patients were different from those whose history of previous suicide attempts was assessed. Actually, we were not able to assess the history of previous suicide attempts only in patients who were early transferred to other PICUs for overcrowding of our PICU or for administrative reasons, i.e. not for clinical reasons. In the emergency psychiatric setting, complete neuropsychiatric assessment of all patients is almost impossible to achieve. The percentage of patients whose history of previous suicide attempts was not assessed was similar in the HCV (16/42; 38.1%) and the non-HCV group (42.0%) [χ^2 ^= 0.123; fd = 1;p = .726]. Third, because so many comparisons were made, significance might be reached by chance in some cases. A cautious interpretation of the marginally significant findings is suggested.

The high rate of HCV infection found in the present study arises concern, especially considering the almost unavoidable chronic course of the infection and its serious consequences. However, the rate is much lower in comparison with the results of previous studies on psychiatric population.

The lower prevalence of HCV infection in our study in comparison with previous studies is probably due to two factors. First, in the emergency psychiatric setting, the HCV prevalence is likely to be lower in comparison with that observed in chronic psychiatric patients. Among a population of Spanish acute psychiatric in-patients, the prevalence of HCV seropositivity was 5.1% [26], a result similar to that observed in the present study. Second, the number of patients with a primary diagnosis of substance-related disorder was relatively low in our sample (52/1492, 3.5%). In Italy, patients with a primary diagnosis of substance-related disorder are treated in specific departments, and not in mental health departments. They are seldom admitted to PICUs. As a consequence, almost only patients with a secondary diagnosis of substance-related disorder entered our study. Consistently with previous studies, we noted a higher frequency of HCV infection in men. However, the difference in gender prevalence failed to reach the statistical significance. In the United States, the rates of hepatitis C infection differ by gender: 2.5% for men compared with 1.2% for women [27]. Among persons with severe mental illness, hepatitis C rates are higher and also differ by gender: 19.6% for men and 9.8% for women [23].

Our results are consistent with the study of Dinwiddie et al. [24] who found that patients positive for hepatitis C virus were more likely to be male, slightly less well educated, and to have a psychoactive substance use disorder diagnosis but no other psychiatric diagnosis. Differently from our study but in accordance with the study of Osher et al [28], they also found that HCV patients were slightly older than the comparison group. However, Osher et al [28] hypothesized that age might not be a risk factor in and of itself but may reflect a complicated cohort effect involving the Vietnam War era. HCV is transmitted primarily through direct percutaneous exposure to blood. Blood transfusion accounted for a substantial proportion of HCV infections before 1990, when routine test began, but now accounts for only a small percentage [17]. Although the main route of transmission is via contaminated blood, in up to 50% of the cases no recognizable transmission factor/route can be identified [15]. While, it is not possible to ascertain the causes of such a high rate, it is tempting to hypothesize that promiscuous and unsafe sexual behavior associated with severe psychiatric illness may be implied as significant risk factor. However, the role of sexual behavior as a risk factor for HCV transmission is still unclear. In a multisite study of blood-borne infections among persons with severe mental illness, the rate of hepatitis C was higher for men at each of the five sites of the study ranging from 7.9% to 35.5%, compared with 4.9% to 16.9% for women [29]. In the same study, sexual risks did not appear to play a major role in hepatitis C transmission since women had significantly more lifetime unprotected sex risks, including vaginal sex, anal sex, sex in exchange for drugs, and sex in exchange for money or gifts. The rate of sexual transmission of hepatitis C virus was found low also in the studies of Tor et al [30] and Fiscus et al [31]. On the other hand, the results of other studies support a more prominent role of sexual transmission of hepatitis C [32-37].

In accordance with previous studies, we found HCV infection related with the diagnoses of any substance-related disorder, alcohol-related disorder, cannabis-related disorder, cocaine-related disorder, opioid-related disorder. The logistic regression confirmed the independent association between any substance-related disorder and HCV infection with a large effect size. While it is easy to attribute the higher prevalence of HCV infection among patients with a opioid-related disorder to their frequent habit of using non sterile needles, it is less clear why the HCV infection is also more prevalent among patients who use only oral or inhalant drugs. HCV prevalence as high as 46% has been found in populations with alcoholic liver disease, even when subject with intravenous drug use and with history of blood transfusion were excluded [38]. Also among alcoholics without liver disease the hepatitis C rate (4.8%) is higher than that in the general population [39] and even tobacco use has been found correlated with the susceptibility to HCV infection [40]. Risky behavior or unknown biological factors associated with substance-related disorder may account for the higher risk of HCV infection in these patients.

Unexpectedly, we found a higher rate of HCV infection among patients with a history of suicide attempt. The logistic regression confirmed the independent association between history of previous suicide attempt and HCV infection. This is the original and major result of the present study which has never been reported before, although depressive symptoms are very frequent during both the acute and chronic stages of hepatitis C [41], and suicidal behavior has been repeatedly reported in patients affected by HCV infection [42]. The result is not easy to explain. Two hypotheses are offered. First, specific treatment of HCV infection, inducing or worsening a depressive state, might have precipitated suicidal behavior. Several reports described suicides or suicide attempts in the course of interferon treatment [43-49]. However, as already noted, no patient had been treated with interferon. Second, HCV infection may be associated with severer forms of psychiatric disorders which carry a higher risk of suicide. The last hypothesis was supported by the findings of worse global functioning in the last year and more hostile or violent behavior during the hospitalization in patients with HCV infection.

## Abbreviations

HCV = Hepatitis C Virus; PICU = Psychiatric Intensive Care Unit; NOS not otherwise specified; BPRS = Brief Psychiatric Rating Scale; GAF = Global Assessment of Functioning; CGI = Clinical Global Impression.
